# Evaluation of IntelliVent-ASV® and PS-SIMV Mode Using Ultrasound (US) Measurements in Terms of Diaphragm Atrophy

**DOI:** 10.7759/cureus.40244

**Published:** 2023-06-11

**Authors:** Gulcin Hilal Alay, Derya Tatlisuluoglu, Guldem Turan

**Affiliations:** 1 Intensive Care Unit, University of Health Sciences, Basaksehir Cam and Sakura City Hospital, Istanbul, TUR

**Keywords:** thickening fraction, ps-simv, mechanical ventilation, diaphragm ultrasound, diaphragm excursion, automated mechanical ventilation

## Abstract

Background: Mechanical ventilation is a life-saving intervention for critically ill patients, but it can also lead to diaphragm atrophy, which may prolong the duration of mechanical ventilation and the length of stay in the intensive care unit. IntelliVent-ASV® (Hamilton Medical, Rhäzüns, Switzerland) is a new mode of ventilation that has been developed to reduce diaphragm atrophy by promoting spontaneous breathing efforts. In this study, we aimed to evaluate the effectiveness of IntelliVent-ASV® and pressure support-synchronized intermittent mandatory ventilation (PS-SIMV) mode in reducing diaphragm atrophy by measuring diaphragm thickness using ultrasound (US) imaging.

Methods: We enrolled 60 patients who required mechanical ventilation due to respiratory failure and were randomized into two groups: IntelliVent-ASV^®^ and PS-SIMV. We measured the diaphragm thickness using US imaging at admission and on the seventh day of mechanical ventilation.

Results: Our results showed that diaphragm thickness decreased significantly in the PS-SIMV group but remained unchanged in the IntelliVent-ASV^®^ group. The difference in diaphragm thickness between the two groups was statistically significant on the seventh day of mechanical ventilation.

Conclusions:IntelliVent-ASV^®^ may reduce diaphragm atrophy by promoting spontaneous breathing efforts. Our study suggests that this new mode of ventilation may be a promising approach to preventing diaphragm atrophy in mechanically ventilated patients. Further studies using invasive measures of diaphragm function are warranted to confirm these findings.

## Introduction

Diaphragm atrophy and dysfunction are clinical outcomes in mechanically ventilated patients [1]. Mechanisms of diaphragm injury have been described as disuse atrophy due to excessive support [2], excessive load due to inadequate support [3], eccentric myotrauma due to diaphragmatic contraction during expiration [4], and asynchronies and longitudinal atrophy due to high positive end-expiratory pressure (PEEP) [5]. Protective mechanical ventilation of the diaphragm has been advocated in recent years, and various studies have been conducted in this regard [6-8]. According to this view, which is also supported by experimental studies, pressure-support ventilation preserves diaphragm protein content and causes less reduction in the cross-sectional area of diaphragmatic muscle fibers compared to controlled mechanical ventilation [9-11].

Ultrasound (US) is a widely used and increasingly popular method in clinical practice for assessing diaphragm damage and detecting changes in diaphragm thickness. Both an increase and a decrease in diaphragm thickness are associated with the outcomes of ventilation [1, 12].

IntelliVent-ASV® (adaptive support ventilation) (Hamilton Medical, Rhäzüns, Switzerland) is a fully automated, closed-circuit ventilation mode that automatically controls gas exchange at the lowest respiratory workload [13] and lowest respiratory effort [14]. This means that both ΔP and mechanical power (MP) are at least partially controlled by IntelliVent-ASV®. Studies have shown that IntelliVent-ASV® reduces delta P and MP [15-17]. IntelliVent-ASV® changes driving pressure, FiO2, and PEEP levels using various equations based on EtCO2 and SpO2, and conducts spontaneous breathing trials at intervals determined by the user.

In our study, we aimed to compare diaphragm thickness measurements in patients using the conventional pressure support mode (PSIMV) and the IntelliVent-ASV® mode.

## Materials and methods

**Figure 1:**
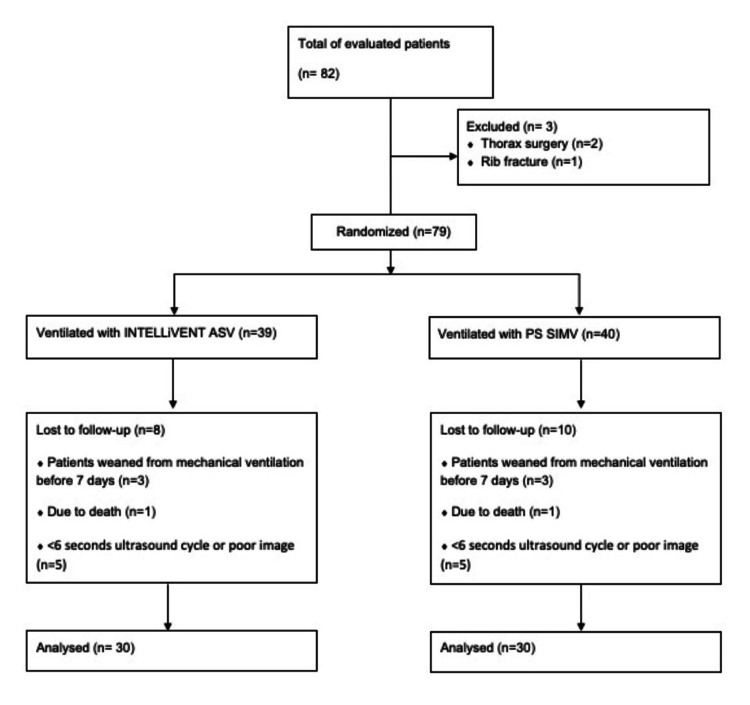
Flow chart.

The study was designed as a prospective single-center randomized study. Ethics committee approval was obtained (KAEK/2022.07.215) and the study adhered to the 2008 Helsinki Declaration. Written informed consent was obtained from the authorized representatives of each patient. Patients over 18 years of age who were intubated were included in the study. A chi-square test will be used to compare the development of diaphragm dysfunction between patients monitored with IntelliVent-ASV® and those monitored with conventional modes. To achieve a power of 80% with an alpha error rate of 0.05 and an effect size of 0.3, it was calculated using G-power 3.1 software that a total of 60 patients needed to be included in the study. According to the principles of randomization, patient enrolment continued until each group reached a total of 30 patients. Exclusion criteria were: (1) age <18; (2) pregnancy; (3) history of pneumothorax, mediastinal emphysema, or chest surgery; (4) flail chest or combined rib fractures; (5) neuromuscular disease; (6) poor image quality or ultrasonography loops less than 6 s; and (7) diaphragm ultrasonography demonstrating the unilateral paradoxical movement of the diaphragm or a history of diaphragmatic paralysis. Patients who had been weaned less than 1 week before and could not undergo the second measurement and those who had undergone mode changes lasting more than 6 h during the study period were excluded from the study (Figure 1).

Patients' age, gender, body mass index (BMI), comorbidities, and APACHE II scores were recorded. The reasons for admission to the ICU were also recorded. Patients were monitored in either IntelliVent-ASV® mode or PS-SIMV mode according to the randomization list. Diaphragm ultrasonography measurements were performed on the first day and seventh day of mechanical ventilation.

Measurements

Diaphragm thickness was measured using the US, following previously described methods [18-20]. A linear array and convex transducer (Arietta 65, Hitachi Ltd., Tokyo, Japan), with frequencies of 13 MHz and 1-5 MHz, respectively, were placed in the ninth or tenth intercostal space near the midaxillary line, angled perpendicular to the chest wall [18]. At this location, the diaphragm appears as a three-layered structure just superficial to the liver, consisting of a relatively non-echogenic muscular layer bounded by the echogenic membranes of the diaphragmatic pleura and peritoneum [21]. Diaphragmatic thickness was measured at end-expiration (TEE) and at peak inspiration (TEI peak thickness value during inspiration) using M-mode, as the distance between the diaphragmatic pleura and the peritoneum [22-23]. TEE and TEI were always measured on two breaths visualized in a single M-mode image. Diaphragm thickening during inspiration was calculated as the difference between TEI and TEE. Diaphragm thickening fraction (TF) was defined as the percentage change in diaphragm thickness during inspiration, computed from the quotient of TEI-TEE and TEE [24]. Delta TEI, TEE, TF, and excursion were calculated as the second measurement minus the first measurement. In addition, the percentage decrease in these measurements and calculations during the first week was also calculated.

Excursion examinations were performed using a 3.5 MHz curvilinear probe. The right hemidiaphragm was first visualized by B-mode, and then M-mode was used to evaluate diaphragmatic excursion during tidal breathing. The right hemidiaphragm was measured by positioning the probe between the midclavicular and midaxillary lines below the right costal margin (subcostal approach), using the liver as an acoustic window. The probe was directed medially, cephalic, and dorsally. When the hemidiaphragm was well visualized, M-mode was applied to measure the excursion. The diaphragmatic excursion was defined as the difference between the highest point and the steep point (amplitude) [25]. Measurement points were marked for the subsequent measurement. One researcher made all of the measurements for the study.

IntelliVent-ASV®

IntelliVent-ASV® is a ventilation mode that comes with adaptive support ventilation (ASV). In both ventilation modes, the clinician enters the patient's height and gender into the ventilator to enable automatic calculation of predicted body weight (PBW). ASV then provides ventilation based on either a minute volume determined by an operator if the patient is passive or based on the patient's demands if they become active or request it. ASV uses the Otis equation to provide the best tidal volume (Vt) and respiratory rate (RR) combination to minimize the work of breathing. Adjustments are made breath by breath, and the ventilator switches from controlled ventilation to supported ventilation or vice versa when the patient becomes active or the minute ventilation becomes too low. IntelliVent-ASV® does the same thing but also uses the Mead equation to adjust Vt, RR, and PEEP to achieve low driving pressure. Additionally, minute ventilation is continuously adjusted based on end-tidal carbon dioxide (etCO2) readings, while PEEP and FiO2 are continuously adjusted based on peripheral oxygen saturation (SpO2) readings. For the latter determinations, IntelliVent-ASV® uses the acute respiratory distress syndrome (ARDS) Network PEEP-FiO2 tables [26-27]. The etCO2 and SpO2 target ranges are partially determined by selecting a lung disorder (acute respiratory distress syndrome and chronic obstructive pulmonary disease) or disease state (brain injury). IntelliVent-ASV® facilitates weaning by gradually reducing minute ventilation and can be set to use a spontaneous breathing trial to reduce ventilator settings within pre-defined limits to identify ready patients, thus covering almost all ventilation aspects from intubation and ventilation initiation to weaning and extubation for passive and active patients. Typical ventilator settings such as VT and airway pressures, respiratory rate, and FiO2 that are typically operator-adjusted and set are continuously adjusted with IntelliVent-ASV® to stay within pre-defined ranges set by the user. It is crucial for the user to input accurate and relevant data, such as the patient's actual height (utilized by the ventilator to estimate the expected body weight and, in turn, regulate VT), as well as the maximum airway pressure (utilized by the ventilator to adjust airway pressure below the set limit). Furthermore, the user may customize the limits of each parameter and may opt to disable one or more of the algorithms employed by IntelliVent-ASV® [28].

Initial ventilator settings were adjusted to FiO2: 50%, PEEP: 6, and the etCO2 target range was set based on the patient's arterial blood gas partial pressure of CO2 at -5 mmHg. The target SpO2 was set at 92%.

PS-SIMV

The pressure support-synchronized intermittent mandatory ventilation (PS-SIMV) mode is suitable for patients requiring prolonged mechanical ventilation as it allows for synchronized spontaneous breathing while preventing respiratory muscle atrophy [29-30]. In the SIMV group, patients were ventilated using the pressure support SIMV mode with pressure support of 10 cmH2O for triggered breaths. The rate of mandatory time-cycled, pressure-controlled breaths was initially set to 12 per minute. Arterial blood gas was obtained at 10-min intervals and every 6 h to adjust the ventilator settings accordingly. Driving pressure maintained under 15 cmH2O.

Statistical analysis

All statistical analyses were performed using SPSS (version 25.0, SPSS Inc., Chicago, IL, USA) and GraphPad Prism (version 8.0, GraphPad Software Inc., La Jolla, CA, USA). Continuous variables were tested for normal distribution using the Shapiro-Wilk test. Normally distributed continuous data were expressed as means ± standard deviation (SD), while non-normally distributed data were presented as medians (interquartile range, IQR). Categorical data were presented as frequencies and percentages. Baseline characteristics and clinical parameters were compared between groups using the independent-samples t-test or Mann-Whitney U test for continuous variables and the chi-square test or Fisher's exact test for categorical variables, as appropriate. Within-group comparisons were performed using the paired-samples t-test or Wilcoxon signed-rank test. Between-group comparisons were made using the unpaired t-test or Mann-Whitney U test. A p-value <0.05 was considered statistically significant. Interobserver variability was analyzed using Bland-Altman plots and the intra-class correlation coefficient (ICC). ICC values >0.75 were considered indicative of excellent agreement. Multiple linear regression analysis was used to identify the factors associated with diaphragm thickness and excursion on day 7. All variables with p <0.1 in the univariate analysis were included in the multivariate analysis.

## Results

The mean age of patients in the IntelliVent-ASV® group was 52.53 ± 18.90, while it was 59.70 ± 11.91 in the PS-SIMV group, and there was no significant difference between the groups (p=0.084). The BMI values were 24.66 ± 3.07 in the IntelliVent-ASV® group and 26.16 ± 5.69 in the PS-SIMV group, and there was no significant difference between the groups (p=0.210). The mean APACHE II scores were 26.15 ± 6.21 in the IntelliVent-ASV® group and 28.21 ± 6.75 in the PS-SIMV group, and there was no significant difference between the groups (p=0.202). The demographic data of the patients are summarized in Table 1. When the indications for ICU admission were categorized into respiratory, sepsis, trauma, and gastrointestinal bleeding, they were found to be 42, 11, 5, and 2, respectively. The ultrasonographic diaphragm measurements (on the first and seventh days of mechanical ventilation) are also provided in Table 1. Table 2 shows the percentage and numerical reductions in diaphragm ultrasonographic measurements on the seventh day of mechanical ventilation. Accordingly, there was a significant reduction in all measurements in the PS-SIMV group compared to the IntelliVent-ASV® group. The large numbers in the standard deviations are due to the fact that the data do not fit the normal distribution and the difference between the minimum-maximum values is large.

**Table 1:** Demographic characteristics and ultrasonographic measurements of the patients. BMI, body mass index; APACHE II, APACHE, acute physiology and chronic health evaluation; CVD, cardiovascular disease; COPD, chronic obstructive pulmonary disease; TEI, thickness at end-inspiration; TEE, thickness at end-expiration; TF, thickening fraction

	IntelliVent-ASV® n=30	PS-SIMV n=30	p
Age	52.53 ± 18.90	59.70 ± 11.91	0.084
BMI	24.66 ± 3.07	26.16 ± 5.69	0.210
APACHE II	26.15 ± 6.21	28.21 ± 6.75	0.202
Gender			
Female	15 (50%)	9 (30%)	0.114
Male	15 (50%)	21 (70%)	
Diabetes mellitus	6 (20%)	13 (43%)	0.052
Hypertension	14 (46.6%)	18 (60%)	0.301
CVD	6 (20%)	10 (33%)	0.243
Arrhythmia	2 (6.6%)	3 (10%)	1.000
Malignancy	9 (30%)	12 (40%)	0.417
COPD	4 (15.3%)	6 (23%)	0.731
TEI (1st day)	2.69 ± 1.03	2.69 ± 1.20	0.947
TEI (7th day)	2.80 ± 0.96	2.19 ± 0.95	0.005
TEE (1st day)	2.10 ± 1.01	2.13 ± 0.99	0.982
TEE (7th day)	2.15 ± 0.97	1.88 ± 0.88	0.090
TF (1st day)	33.93 ± 17.90	27.01 ± 12.93	0.183
TF (7th day)	34.44 ± 16.73	17.26 ± 8.27	<0.001
Excursion (1st day)	17.30 ± 7.34	13.63 ± 4.74	0.045
Excursion (7th day)	17.60 ± 7.64	10.77 ± 3.74	0.001

**Table 2:** Comparison of percentage and numerical values of decrease in measurements between groups. TEI, thickness at end-inspiration; TEE, thickness at end-expiration; TF, thickening fraction; SD, standard deviation

	IntelliVent-ASV^®^ Mean ± SD N=30	PS-SIMV Mean ± SD N=30	p
Delta TEI (mm)	-0.11 ± 0.40	0.50 ± 0.58	<0.001
TEI change (%)	-6.76 ± 19.77	16.76 ± 12.19	<0.001
Delta TF (mm)	-0.51 ± 13.17	9.75 ± 8.14	<0.001
TF change (%)	-5.50 ± 34.91	32.22 ± 23.36	<0.001
Delta excursion (mm)	-0.30 ± 2.13	2.85 ± 2.53	<0.001
Excursion change (%)	-1.53 ± 11.32	19.45 ± 15.96	<0.001

## Discussion

As far as we know, this is the first study to compare an automated mechanical ventilation mode with a conventional supported mode in terms of diaphragm US measurements. The most important finding was that there was significantly less thinning in diaphragm US measurements in patients ventilated with Intellivent ASV than with conventional ventilation. In a randomized controlled trial in patients who underwent orthotopic liver transplantation comparing adaptive support ventilation (ASV) with PSIMV mode, patients who followed with the ASV mode in the postoperative period were observed to be weaned earlier. Since the intubation periods in this study were postoperative patients, they were short [31]. In our study, diaphragm US measurements were performed in patients with long mechanical ventilation periods. It is generally difficult to evaluate the effect of different ventilation modes on clinical outcomes. Therefore, we do not have a primary endpoint such as weaning or mortality. In our study, we used the IntelliVent-ASV® mode, which uses the same formulas as ASV, but adjusts the pressure support level, tidal volume, and respiratory rate according to EtCO2. Intellivent ASV also adjusts PEEP and FiO2 levels according to SpO2 values and is a mode that automatically performs spontaneous breathing trials. Therefore, we started with the hypothesis that the diaphragm muscle could undergo less atrophy in the IntelliVent-ASV® mode, which allows more spontaneous breathing trials, prevents volutrauma and barotrauma due to self-control, and also prevents hyperoxia. It is believed that the fact that the control group is not in a controlled ventilation mode but a weaning mode, PSIMV mode, will increase the power of the study.

Lixian et al. showed that after the combination of SIMV with AutoFlow, the peak inspiratory pressure and mean airway pressure significantly decreased, and the airway resistance decreased; this shows that AutoFlow can improve respiratory mechanics to some extent [32]. The working principle of AutoFlow is as follows: at the beginning of the ventilator, four consecutive experimental breaths with a pressure of 10 cm H2O are performed, and the current airway pressure and lung-chest compliance of the patient are continuously measured by a microcomputer. According to the volume-pressure relationship, the suction pressure required for the next ventilation to reach the preset tidal volume is calculated, the preset suction pressure level is automatically adjusted (usually adjusted to 75% of the calculated value), and the preset tidal volume is sent with the velocity waveform of deceleration wave and the lowest pressure so that the actual tidal volume is consistent with the preset tidal volume and air pressure injury is avoided to the greatest extent [32]. However, AutoFlow itself is not an independent ventilation mode. IntelliVent-ASV® is also one of the auto modes and can be used as a single mode. Auto modes are newly developed modes aimed at reducing clinician-dependent ventilator-associated lung injury and their use is becoming more common.

In a study conducted on COPD and healthy volunteers, as the degree of dynamic hyperinflation increased, the TF decreased significantly [33]. We believe that the tidal volume and pressure support changes of IntelliVent-ASV® according to EtCO2 can reduce dynamic hyperinflation, but clinical and experimental studies need to be conducted on the subject. In studies evaluating those with and without sepsis on the subject, the TF was significantly higher in non-septic patients [34-36]. However, these studies were single-center and emphasized the necessity of conducting multicenter studies on the subject. Our study involved a heterogeneous patient group consisting mainly of patients in the intensive care unit due to respiratory diseases.

The accuracy of ultrasonography can be influenced by several factors such as the operator's skills and experience, the angle of incidence of the US beam, and the quality of the imaging. The diaphragm is a thin muscle with an average thickness of only 1.7-2 mm when measured at the apposition zone in healthy subjects [37], and it can become even thinner during mechanical ventilation [12, 38-39]. Therefore, accurately measuring diaphragm thickness using the US can be challenging [40]. Goligher and colleagues confirmed that errors can occur in measuring diaphragmatic US data [12].

The study has several limitations. First, only the right diaphragm was analyzed to minimize volunteer pressure and effort. Second, no invasive gold standard measurements, such as trans diaphragmatic pressure or diaphragm electrical activity, were performed. Third, other possible causes of diaphragm dysfunction, aside from those listed in the exclusion criteria, were not taken into account. Other conditions that may lead to diaphragm dysfunction, such as nutritional status, corticosteroid use, sedation, and muscle relaxant use, were not taken into account. The other causes of diaphragm dysfunction include residual sedation or simply lack of effort in required ventilation [41-42].

However, our study has several strengths. The measurements were conducted by a single practitioner. The measurement site was marked, and the measurement was repeated by the same practitioner in the same position from the same area after 7 days. A study investigating the repeatability of diaphragm US emphasized that the measurement was appropriate and repeatable for monitoring diaphragm activity and function [12].

## Conclusions

IntelliVent-ASV® is a new auto-mode ventilation system. It is expected to reduce diaphragm atrophy due to its high patient-ventilator synchrony and ability to perform spontaneous breathing trials. Our study demonstrated a reduction in diaphragm atrophy with the use of diaphragm US. However, future studies using gold-standard methods for measuring diaphragm activity will provide further clarity on the subject.
